# Fecal *Helicobacter pylori* PCR: what agreement with urea breath test does not prove

**DOI:** 10.1128/jcm.01164-26

**Published:** 2026-09-17

**Authors:** Christos Zavos

**Affiliations:** 1 Department of Gastroenterology, Bioclinic Hospital of Thessaloniki, Thessaloniki, Greece; Department of Medicine, Maine Medical Center, Portland, Maine, USA

**Keywords:** urea breath test, fecal PCR, *Helicobacter pylori*

## LETTER

I read with interest the paper by Wang et al. (1) who reported near-perfect agreement between a 16S ribosomal RNA (rRNA)-targeted fecal PCR assay and the ^13^C-urea breath test (UBT) in 777 patients in 4 hospitals. As a clinical gastroenterologist, my concern begins after the result reaches my hands, and I hope the authors can help me make better decisions based on their data.

Their assay tells the clinician that *Helicobacter pylori* (*H. pylori*) is present, while it says nothing about which drug will eradicate it. That gap matters more in China than in most places: in the CHINAR-MAP surveillance, clarithromycin resistance was predicted in 1,776 of 3,494 infected people screened in 52 cities (2). Maastricht VI and the 2024 American College of Gastroenterology guideline now reserve clarithromycin for strains shown to be susceptible (3, 4), and in a stool PCR cohort published in this journal, triple therapy eradicated the infection in 41% of patients with predicted resistance against 70% of the others (*P* = 0.03) (5). Pooled randomized data put eradication at 85% with susceptibility-guided regimens and 75% with empiric ones (6). What puzzles me is that in the Introduction, the authors offer simultaneous detection of the A2143G mutation as a reason to move to fecal PCR, and in the Discussion, they count resistance detection among the advantages, yet neither was evaluated. Had 23S rRNA been chosen as the target, one reaction would have returned both answers (7). Applying the national figure to the 341 positives, roughly 20% of those tested would be expected to carry a resistant strain this assay could not see. Is the extracted DNA still stored, and could the resistance prevalence of the cohort be reported?

As regards cost and setting, the comparator was a second PCR kit rather than the monoclonal stool antigen test that guidelines accept for diagnosis and for confirmation of cure and that needs no thermal cycler (3, 4). Bead beating, column purification, storage at −80°C, and amplification within 72 h are not trivial in the settings the authors have in mind. Radiation does not decide the question either, since the reference test was the ^13^C-UBT and ^13^C is stable (7). My question is, were antigen results obtained in any subset, and what did one test cost at each site?

Finally, the 94.7% sensitivity after eradication was attributed to detection of residual DNA, but residual DNA makes PCR positive when UBT is negative, which is the opposite discordance, and the review cited treats it as a post-treatment false positive (8). Eighteen of 19 patients (95% confidence interval 73.97–99.87) cannot settle the question in either direction. Ct values from those discordant post-treatment samples would show whether a low bacterial load or residual DNA from a cured *H. pylori* infection was being detected.

I would appreciate the authors’ input on the above thoughts.

## EDITOR'S NOTE

**Ed. Note**: The authors of the original article declined to respond.
